# Patterns of Immune Infiltration in HNC and Their Clinical Implications: A Gene Expression-Based Study

**DOI:** 10.3389/fonc.2019.01285

**Published:** 2019-12-04

**Authors:** Jukun Song, Zhenghao Deng, Jiaming Su, Dongbo Yuan, Jianguo Liu, Jianguo Zhu

**Affiliations:** ^1^Department of Oral and Maxillofacial Surgery, Guizhou Provincial People's Hospital, Guiyang, China; ^2^School of Medicine, Guizhou University, Guiyang, China; ^3^Department of Pathology, School of Basic Medicine, Central South University, Guangzhou, China; ^4^Department of Urology, Guizhou Provincial People's Hospital, Guiyang, China; ^5^Department of Oral Medicine, School of Stomatology, Zunyi Medical University, Zunyi, China

**Keywords:** immune infiltration, head and neck cancer, tumor microenvironment, CIBERSORT, TCGA

## Abstract

**Background:** Immune infiltration of head and neck cancer (HNC) highly correlated with the patient's prognosis. However, previous studies failed to explain the diversity of different cell types that make up the function of the immune response system. The aim of the study was to uncover the differences in immune phenotypes of the tumor microenvironment (TME) between HNC adjacent tumor tissues and tumor tissues using CIBERSORT method and explore their therapeutic implications.

**Method:** In current work, we employed the CIBERSORT method to evaluate the relative proportions of immune cell profiling in 11 paired HNC and adjacent samples, and analyzed the correlation between immune cell infiltration and clinical information. The tumor-infiltrating immune cells of TCGA HNC cohort was analyzed for the first time. The fractions of LM22 immune cells were imputed to determine the correlation between each immune cell subpopulation and survival and response to chemotherapy. Three types of molecular classification were identified via “CancerSubtypes” R-package. The functional enrichment was analyzed in each subtype.

**Results:** The profiles of immune infiltration in TCGA HNC cohort significantly vary between paired cancer and para-cancerous tissue and the variation could reflect the individual difference. Total Macrophage, Macrophages M0 and NK cells resting were elevated in HNC tissues, while total T cells, total B cells, T cells CD8, B cell navie, T cell follicular helper, NK cells activated, Monocyte and Mast cells resting were decreased when compared to paracancerous tissues. Among each cell immune subtype, T cells regulatory Tregs, B cells naïve, T cells follicular helper, and T cells CD4 memory activated was significantly associated with HNC survival. Three clusters were observed via Cancer Subtypes R-package. Each cancer subtype has a specific molecular classification and subtype-specific immune cell characterization.

**Conclusions:** Our data suggest a difference in immune response may be an important driver of HNC progression and response to treatment. The deconvolution algorithm of gene expression microarray data by CIBERSOFT provides useful information about the immune cell composition of HNC patients.

## Introduction

Head and neck cancer (HNC) remain the primary cause of cancer-related mortality in the world. Oral squamous cell carcinoma (OSCC) is the most important type of HNC, accounting for about 90%, characterized by advanced diagnosis (1), poor prognosis (2), low overall survival (3), and high recurrence (4). Head and neck cancer (HNC) are regarded as the tenth most common cancer in the world and the seventh most common cause of cancer deaths. There are ~400,000 oral and pharyngeal diseases, 160,000 laryngeal cancers and 300,000 deaths worldwide each year (5–7). Head and neck cancer is a common heterogeneous tumor that exists in the oral, pharyngeal, and larynx lesions (8). Contributing factors may include fewer mutant oncogenes, the presence of extensive p53 and lower levels of tumor hypoxia, but the mechanisms remain to be explored in detail. The development of cancer genomics has been widely used to reveal subgroups of patients with different prognoses and different responses to treatment. However, the phenotype of cancer is defined not only by the intrinsic activity of tumor cells but also by immune cells recruited and attracted in the tumor-associated microenvironment. Until now, the roles of immune cells in the tumor-related microenvironment during tumor development remain unclear.

There is increasing evidence that tumor cell immune cells (TIICs), which including B cells, T cells, dendritic cells, macrophages, neutrophils, monocyte, and mast cells, can regulate cancer progression and stimulate attractive therapeutic targets. In addition, the effect of TIICs on cancer development has been largely documented (9–12). In the work, the CIBERSOFT method was applied to define the 22 TICCs subsets of the immune response in HNC in order to examine the mutual relationship with molecular subsets and clinical characteristics. Our findings also revealed distinct immune phenotypes for molecular HNC subclasses. Moreover, the present study provides a novel insight into immunotherapy strategy for HNC.

## Materials and Methods

### Data Source and Data Processing

Firstly, the RNA-seq data (FPKM values) of HNC cohort were downloaded from the Cancer Genome Atlas (TCGA) website (https://cancergenome.nih.gov/), including four files (gdc_download_20190201_030206.452249.tar.gz, gdc_manifest_20190201_025835.txt, metadata.cart.2019-02-01.json and clinical.cart.2019-02-01.json). Secondly, we merged the data using Perl software (mRNA_merge.pl). RNA-seq profiles were normalized using Voom standardized method (variance modeling at the observational level) (13).

### Quantification of TIICs Using the CIBERSOFT Algorithm

The CIBERSOFT method is a gene-based deconvolution algorithm that infers 22 human immune cell types and uses the characteristics of 547 marker genes to quantify the relative scores for each cell type. To enhance the robustness of the results, CIBERSOFT algorithm uses Monte Carlo sampling to derive the deconvoluted P value for each sample. The standardized processed dataset of gene expression is uploaded to the CIBERSOFT website (https://cibersort.stanford.edu/index.php), which runs using 1000 aligned default signature matrices (14).

Total T cells were calculated as a sum of CD8+ T cells, CD4+ naïve T cells, CD4+ memory resting T cells, CD4+ memory activated T cells, follicular helper T cells, regulatory T cells (Tregs), and T cells gamma delta fractions. Total macrophage fraction was imputed as a sum of M0, M1, and M2 macrophage fractions. And total B cells was estimated as a sum of B cells memory and B cells naïve.

### Survival Analysis

We conducted univariate Cox analysis and Kaplan-Meier survival analysis between LM22 immune cell subsets and OS and screened the prognostic 22 human immune cell phenotypes. The univariate Cox analysis and Kaplan-Meier survival analysis was performed by the survfit function in “survival” package in R software. Then, the multivariate Cox regression analysis was used to further validate prognostic 22 human immune cell phenotypes as prognosis factors. We also explore the association between LM22 immune cell and other clinical information, such as TNM stage, Radiation, Grade, Age, and HPV status.

### Identification of Molecular Subclassification

In order to further to explore different TME cell infiltration patterns, a consensus cluster algorithm was applied to determine the number of clusters. The process was conducted using “CancerSubtypes” R-package (15). To uncover the potential difference in TME cell infiltration models among the clusters, differentially expressed genes (DEGs) and different immune cell types were identified using unpaired Student *t* tests. The data set with |log2 fold change| ≥ 0.2 and *P* –value less than 0.05 was considered selection criteria for subsequent analysis.

### Functional and Pathway Enrichment Analysis

To uncover the potential biological significance of DEGs among TME subtypes, Gene Ontology (GO) Biological Process term and Kyoto Encyclopedia of Genes and Genomes (KEGG) pathway analysis were conducted using “ClusterProfiler” R package (16). GO enrichment analysis was based on the threshold of *P*-value < 0.05 and false discovery rate (FDR) of < 0.05. Gene Set Variation Analysis (GSVA) was conducted to unveil an overall pathway of gene-set activity score for each sample (17). The Gene sets using the c2 curated signatures were downloaded from the Molecular Signature Database (MSigDB) of Broad Institute. The significant enrichment pathway was identified based on the |logFoldChange| ≥0.2 and adjust *P*-value < 0.05.

### Statistical Analysis

Patients with a CIBERSOFT P value less than 0.05 were included in the main study. After the initial screen, eligible samples were divided into two groups: HNC tumor samples and adjacent samples. Immune cell profiles were estimated for each sample. In order to explore the association among 21 immune cell subtypes in three groups, Pearson correlation analysis was calculated using R software. The wilcox.test was employed to test the statistical significance between the two groups. For comparisons of more than two groups, Kruskal-Wallis tests were employed to examine the difference among groups (18). Cox regression analysis was applied to determine the association between inferred proportions of immune cell types and survival. The log-rank statistic was used to test the differences in OS survival between groups using Kaplan-Meier plots. In the univariate Cox regression analysis, variables with a *P* < 0.05 were considered as independent prognostic overall survival (OS) factors, and the included prognostic factors were used to build the multivariate Cox regression model for OS. Clinical variables, such as age, sex, HPV status, lymph node metastasis, distant metastasis, grade, and TNM stage, were included in the multivariate Cox regression model. To evaluate the relationship between different immune cell subtypes and response to radiation, the wilcox.test was conducted.

A heatmap was produced with the R package “ComplexHeatmap” (19). The R package “pROC” was used to visualize operating characteristic (ROC) curves to impute the area under the curve (AUC) and confidence intervals to evaluate the diagnostic accuracy of LM 22 immune cell (20). Statistical analysis was performed using R-Language (R-project.org) and packages obtained through the Bioconductor project (www.bioconductor.org). All *P* values were bilateral and a *P* value of < 0.05 was considered statistically significant.

## Results

### Overview of Data

A total of 546 samples, included 44 adjacent samples, and 502 tumor samples, were obtained from the TCGA. After performing CIBERSOFT algorithm, 454 patients (11 normal patients and 443 tumor patients) with a *P* value < 0.05 was considered in the study, including 41 paracancerous tissue, and 11 paired tumor tissue. Meanwhile, 547 TME corresponding gene expression profiles were also filtered for further analysis.

### Profile of TME in HNC and Clinicopathological Characteristics of TME Subtypes

**Figure 1:**
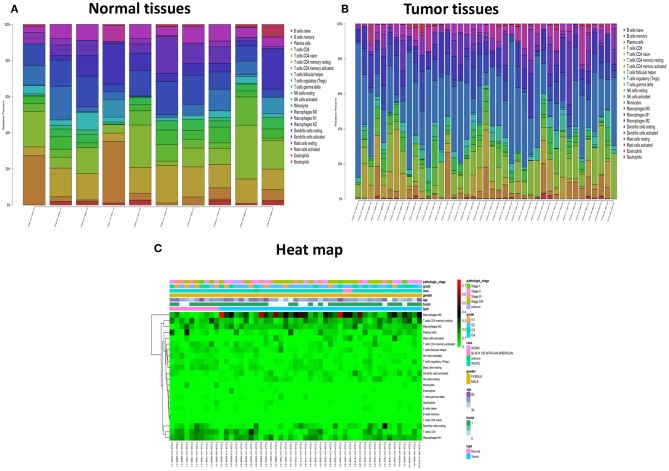
The performance of CIBERSOST for estimating TIICs composition in HNC. **(A)** Relative proportions of 22 TIICs subpopulation in normal samples. **(B)** Relative proportions of 22 TIICs subpopulation in tumor samples. **(C)** Heat map of the 22 immune cell proportions.

**Figure 2:**
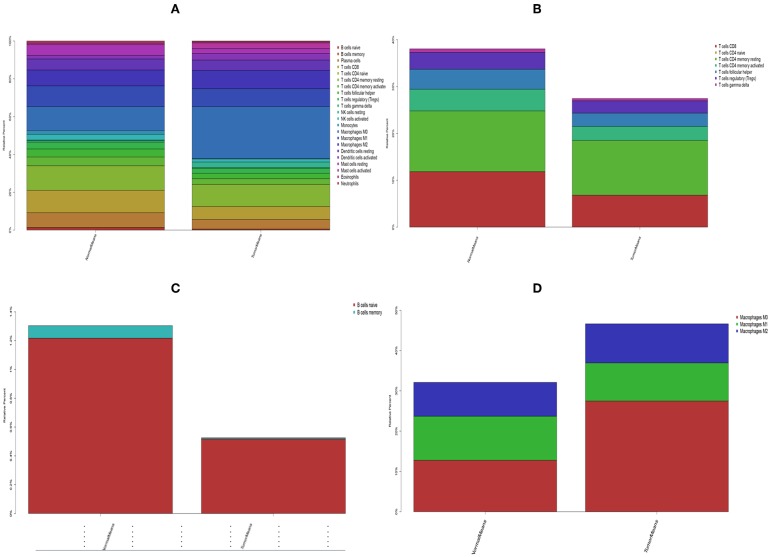
The stacked histogram shows the distribution of 22 immune cell infiltration between HNC adjacent tissues and tumor tissues, including total immune cells **(A)**, total T cells **(B)**, total B cells **(C)**, and total macrophages **(D)**.

**Figure 3:**
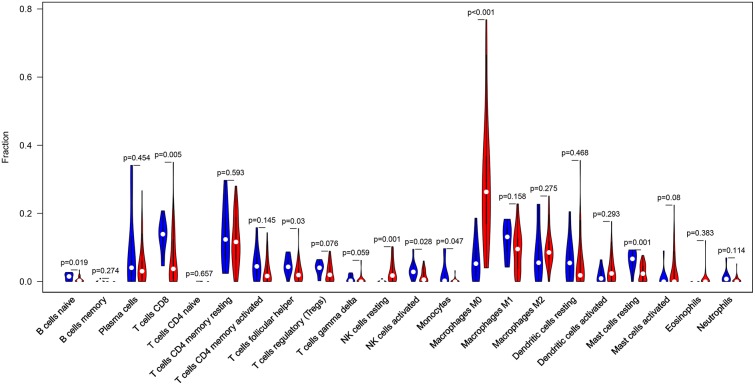
The Violin plot exhibits the difference between CIBERSOFT immune cell fractions between HNC adjacent tissues and tumor tissues.

The landscape of TME cell infiltration models and MTE signatures was systematically evaluated by CIBERSOFT algorithm. Figure 1 summarizes the findings obtain from the 41 paired tumor samples and 11 paired adjacent samples. Obviously, the proportions of TME cells in HNC varies significantly between both intra- and intergroup. The fraction of total T cells and total B cells were higher in HNC adjacent tissue than tumor tissue. Total Macrophage was mainly observed in the adjacent tissue (Figure 2). We found that T cell follicular helper, T cells CD8, NK cells activated, Monocyte, Macrophages M0, NK cells resting, Mast cells resting, and B cell navie were significantly changed between normal and tumor group, while B cells memory, Plasma cells, T cells CD4 naive, T cells CD4 memory resting, T cells CD4 memory activated, T cells regulatory (Tregs), T cells gamma delta, Macrophages M1, Macrophages M2, Dendritic cells resting, Dendritic cells activated Eosinophils, and Neutrophils were not obviously altered between groups (Figure 3, Table 1).

**Table 1:** Comparison of CIBERSORT immune fractions between HNC tumor tissues and adjacent tissues.

**Immune cells subtype**	***P* value**	**Normal means**	**Tumor means**	**logFC**
B cells naive	0.019	0.012169	0.005176	−1.2333
B cells memory	0.274	0.000872	8.02E-05	−3.4427
Plasma cells	0.454	0.078889	0.051193	−0.62387
T cells CD8	0.005	0.118383	0.0682	−0.79561
T cells CD4 naive	0.657	0	2.15E-05	Inf
T cells CD4 memory resting	0.593	0.129735	0.116975	−0.14936
T cells CD4 memory activated	0.145	0.046328	0.02998	−0.62785
T cells follicular helper	0.03	0.042598	0.028291	−0.59047
T cells regulatory (Tregs)	0.076	0.0362	0.02617	−0.46804
T cells gamma delta	0.059	0.007368	0.00496	−0.57091
NK cells resting	0.001	0.003734	0.02877	2.94596
NK cells activated	0.028	0.030262	0.015923	−0.92639
Monocytes	0.047	0.019185	0.002836	−2.75803
Macrophages M0	<0.001	0.127735	0.274906	1.10578
Macrophages M1	0.158	0.109336	0.094605	−0.20877
Macrophages M2	0.275	0.084117	0.097047	0.20629
Dendritic cells resting	0.468	0.058007	0.054292	−0.0955
Dendritic cells activated	0.293	0.019029	0.034497	0.858275
Mast cells resting	0.001	0.056576	0.026052	−1.11881
Mast cells activated	0.08	0.008166	0.030253	1.889414
Eosinophils	0.383	0.000171	0.00494	4.851694
Neutrophils	0.114	0.011142	0.004832	−1.20542

**Figure 4:**
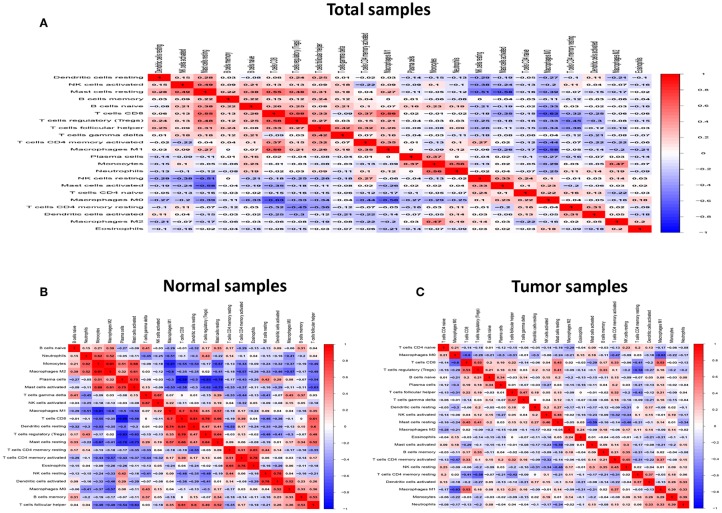
Correlation matrix of all 22 immune proportions and immune cytolytic activity in the TCGA HNC cohort, including total samples **(A)**, normal samples **(B)** and tumor samples **(C)**.

Form the Figure 4, we can see that several highly positive relationship among LM22 immune cells in the HNC paired adjacent tumor samples were found, while mutual relationship among LM22 immune cells reduced in the tumor samples, such as Macrophages M2 was highly positive associated with Monocytes in the immune phenotype profiles in the TMC form the HNC paired adjacent tumor samples, while T cells regulatory (Tregs) was highly negatively related to T cells CD8 in the HNC tumor group. Thus, we hypothesized that alterations in the TME cell infiltration ratio directly reflect the difference in immunity between the two groups.

**Figure 5:**
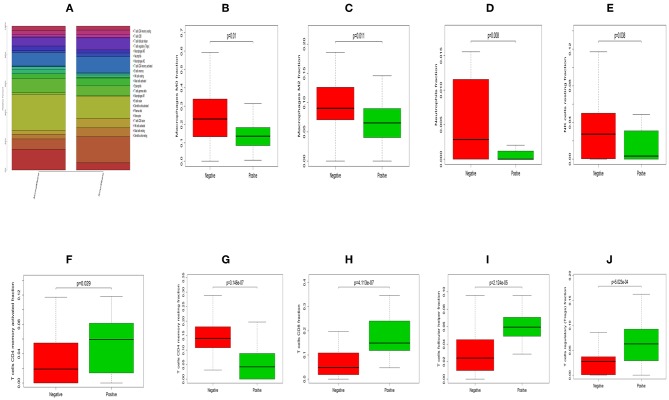
The difference between immune infiltration between HNC HPV positive and negative samples. **(A)** The total difference in immune cells infiltration. Box plot of the distribution of CIBERSORT *P* value for Macrophages M0 **(B)**, Macrophages M2 **(C)**, Neutrophils **(D)**, NK cells resting **(E)**, T cells CD4 memory activated **(F)**, T cells CD4 memory resting **(G)**, T cells CD8 **(H)**, T cells follicular helper **(I)** and T cells regulatory (Tregs) **(J)**, respectively.

In terms of clinical features, we can see that the fraction of T cells CD8, T cells follicular helper, T cells regulatory (Tregs) and T cells CD4 memory activated was higher in HNC HPV positive tissue than HNC HPV negative tissue, while NK cells resting, Macrophages M2, T cells CD4 memory resting, Macrophages M0 and Neutrophils were higher in the HNC HPV negative samples (Figure 5, Table 2). T cells follicular helper was mainly observed in the early stage (G1/G2) of HNC (Supplementary Figure 1, Supplementary Table 1). Neutrophils and Macrophages M1 was associated with radiation therapy (Supplementary Figure 2, Supplementary Table 2). Therefore, these results demonstrated that aberrant immune infiltration and it's heterogeneous in HNC as a tightly regulated process might play important roles in the development of tumor, it has important clinical meanings.

**Table 2:** Comparison of CIBERSORT immune fractions between HNC HPV positive patients and negative patients.

**Immune cells**	**Normal means**	**Tumor means**	**logFC**	***P* Value**
B cells memory	2.17E−05	0.004647	7.741616	0.000231
B cells naive	0.014956	0.024706	0.724158	0.518213
Dendritic cells activated	0.03191	0.021905	−0.54275	0.065003
Dendritic cells resting	0.031514	0.02751	−0.19604	0.891241
Eosinophils	0.001891	0.000123	−3.94557	0.718216
Macrophages M0	0.247879	0.154108	−0.6857	0.009724
Macrophages M1	0.091053	0.110677	0.281571	0.359036
Macrophages M2	0.097167	0.069925	−0.47465	0.011035
Mast cells activated	0.020716	0.008805	−1.23435	0.02111
Mast cells resting	0.027321	0.031731	0.215875	0.178435
Monocytes	0.003497	0.001689	−1.04969	0.88545
Neutrophils	0.013373	0.001426	−3.22932	0.007963
NK cells activated	0.015302	0.020886	0.448863	0.179469
NK cells resting	0.0288	0.017236	−0.74063	0.038035
Plasma cells	0.060632	0.083056	0.454019	0.184296
T cells CD4 memory activated	0.034592	0.053941	0.640956	0.028705
T cells CD4 memory resting	0.144439	0.050991	−1.50214	3.15E-07
T cells CD4 naive	1.00E-05	0	Inf	0.563299
T cells CD8	0.071886	0.181113	1.3331	4.11E-07
T cells follicular helper	0.031526	0.061919	0.973848	2.12E-05
T cells gamma delta	0.003494	0.007961	1.187876	0.002974
T cells regulatory (Tregs)	0.028021	0.065644	1.228147	0.000502

### Identification of Prognostic LM22 Immune Cell Subtypes

**Figure 6:**
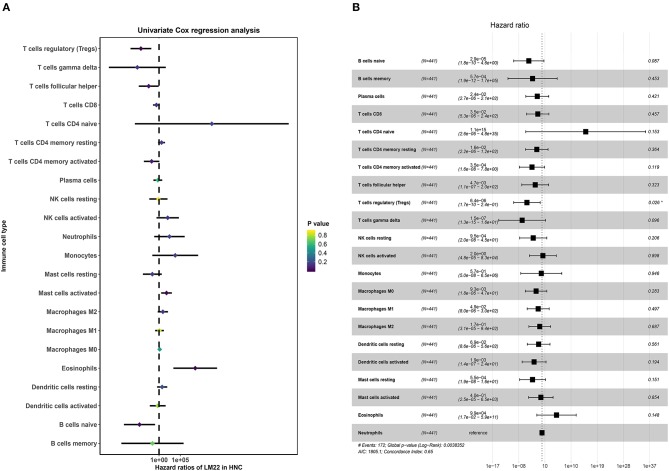
The prognostic associations of subsets of immune cells in Cox regression analysis. **(A)** Univariate Cox regression analysis. **(B)** Multivariate Cox regression analysis.

**Figure 7:**
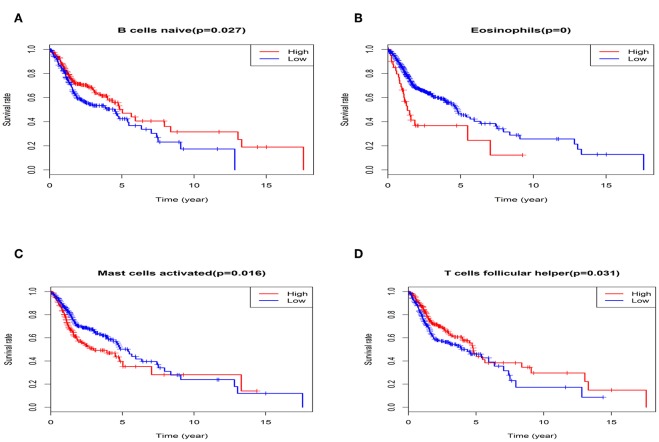
The K-M survival analysis of four immune cells, including B cells naïve **(A)**, Eosinophils **(B)**, Mast cells activated **(C)**, and T cells follicular helper **(D)**.

The univariate Cox regression was conducted to screen the prognostic LM22 immune cell subsets in all tumor samples, and the results were shown that a total of 6 immune cell subsets (Eosinophils, T cells regulatory Tregs, Mast cells activated, B cells naïve and T cells follicular helper) was significantly correlated with OS when the *P*-value was less than 0.05 (Figure 6A). B cells naive, T cells regulatory (Tregs), T cells follicular helper were associated with improved outcome. Mast cells activated and Eosinophils were associated with poorer outcome. Then, we conducted a Kaplan-Meier curve plot and log-rank test for the above-mentioned immune cell subsets, and the results are shown in Figure 7. Eosinophils, Mast cells activated, B cells naïve and T cells follicular helper were significantly associated with OS with HNC patients.

**Figure 8:**
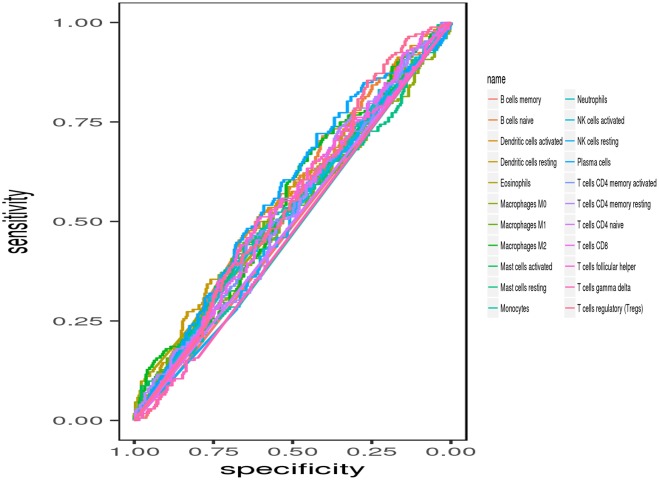
ROC curves of prognostic predictors built by the 22 immune cell subsets.

Next, multivariate Cox proportional hazards regression analysis was further identified the prognostic LM22 immune cell subsets. The results indicated that T cells regulatory (Tregs) was associated with improved outcome (Figure 6B). The ROC curves were used to evaluate the prognostic power of prognostic LM22 immune cell subsets. The AUC for prognostic LM22 immune cell subsets biomarkers prognostic model was shown in Figure 8. In HNC, Dendritic cells activated, B cells naïve, Dendritic cells resting, Macrophages M0, Macrophages M2, Plasma cells, and T cells follicular helper had an AUC value of more than 0.550, and T cells follicular helper had the highest performance in the risk prediction of HNC patients. Detailed results are provided in Table 3.

**Table 3:** The AUC value for each immune cell.

**Immune cells subtype**	**AUC**	**AUC; 95%CI**
B cells naive	0.568	0.503–0.628
B cells memory	0.489	0.283–0.856
Plasma cells	0.571	0.515–0.623
T cells CD8	0.534	0.478–0.588
T cells CD4 naive	0.498	0.102–0.966
T cells CD4 memory resting	0.534	0.479–0.587
T cells CD4 memory activated	0.532	0.472–0.590
T cells follicular helper	0.600	0.490–0.601
T cells regulatory (Tregs)	0.548	0.493–0.604
T cells gamma delta	0.477	0.368–0.684
NK cells resting	0.510	0.449–0.568
NK cells activated	0.525	0.472–0.606
Monocytes	0.484	0.37–0.583
Macrophages M0	0.551	0.449–0.560
Macrophages M1	0.532	0.480–0.585
Macrophages M2	0.550	0.501–0.612
Dendritic cells resting	0.551	0.494–0.605
Dendritic cells activated	0.553	0.491–0.612
Mast cells resting	0.520	0.447–0.578
Mast cells activated	0.536	0.463–0.624
Eosinophils	0.535	0.283–0.844
Neutrophils	0.534	0.465–0.600

**Figure 9:**
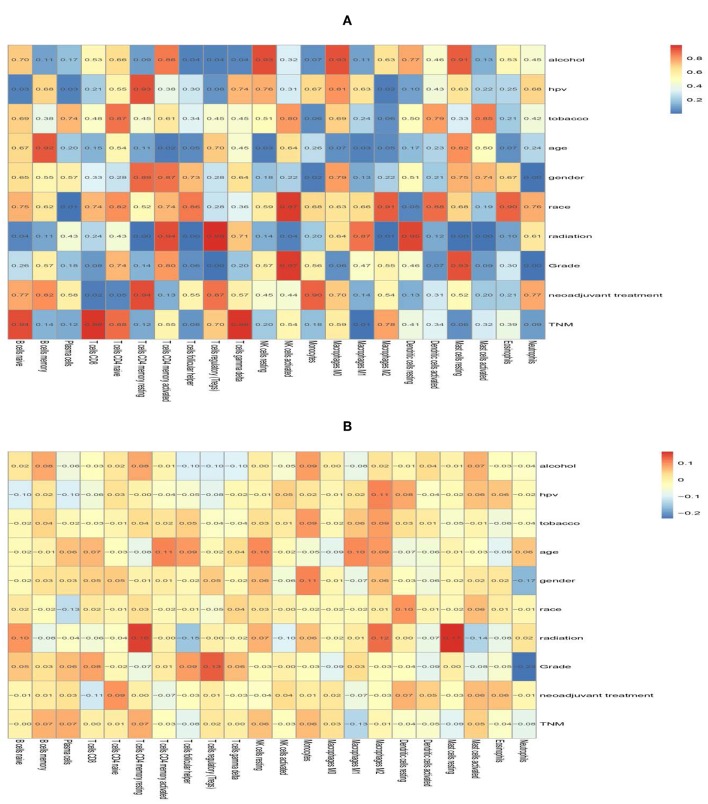
The correlation heat map of 22 immune cells and clinical features. The value in the space shows the correlation coefficient **(A)** and the *P* value **(B)**.

**Figure 10:**
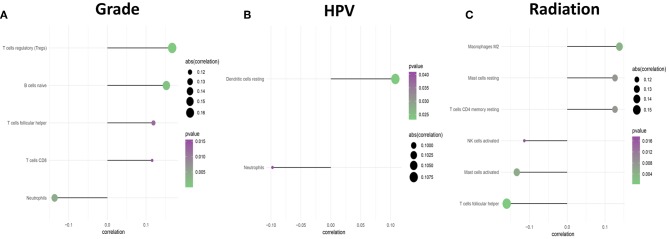
The forest plots show the correlation between clinical characteristics and immune cell subsets. **(A)** Is Grade, **(B)** is for HPV status, and **(C)** is for Radiation.

Subsequently, the association between clinical features and LM22 immune cell subsets was performed to confirm the mutual relationship. After analysis, clinical covariates of HPV status, and gender, grade, race, radiation, age were significantly correlated with immune cell subclasses. However, clinical covariates of alcohol consumption, tobacco consumption, TNM stage, and neoadjuvant treatment are not significantly correlated with LM22 immune cell subsets (Table 4, Figure 9). Among these clinical variables, radiation/grade/HPV was significantly associated with immune cell subclasses (Figure 10).

**Table 4:** Differences in pathway activities scored per cell by GSVA between HNC tumor and normal tissues.

**Kegg pathway**	**logFC**	**AveExpr**	***t***	***P* Value**	**adj.P.Val**	**B**
HALLMARK_APICAL_JUNCTION	−0.4025	0.010961	−5.25587	2.32E-07	6.26E-06	6.544336
HALLMARK_HEME_METABOLISM	−0.28374	−0.00808	−4.51909	8.03E-06	0.000108	3.184278
HALLMARK_XENOBIOTIC_METABOLISM	−0.43824	0.012682	−4.39633	1.39E-05	0.000125	2.668925
HALLMARK_KRAS_SIGNALING_DN	0.238528	0.025281	4.240365	2.73E-05	0.000184	2.032894
HALLMARK_ALLOGRAFT_REJECTION	0.213846	0.003377	4.134352	4.28E-05	0.000231	1.612623
HALLMARK_TNFA_SIGNALING_VIA_NFKB	−0.22983	−0.01298	−3.52174	0.000474	0.001829	−0.6217
HALLMARK_KRAS_SIGNALING_UP	−0.18953	0.007866	−3.36564	0.000832	0.002807	−1.13723
HALLMARK_FATTY_ACID_METABOLISM	−0.30329	0.013411	−3.11011	0.001994	0.005981	−1.93318
HALLMARK_INTERFERON_GAMMA_RESPONSE	0.175599	0.015616	2.771092	0.005828	0.015735	−2.89629
HALLMARK_EPITHELIAL_MESENCHYMAL_TRANSITION	−0.22674	0.017215	−2.18466	0.02945	0.06824	−4.30808
HALLMARK_IL2_STAT5_SIGNALING	0.116782	0.007269	2.17291	0.030329	0.06824	−4.33303
HALLMARK_APICAL_SURFACE	0.182865	0.000551	2.049396	0.041026	0.085208	−4.58742
HALLMARK_SPERMATOGENESIS	−0.13185	0.012642	−1.90605	0.057306	0.110518	−4.86433
HALLMARK_ESTROGEN_RESPONSE_EARLY	−0.19236	0.023794	−1.72959	0.084417	0.15195	−5.1781
HALLMARK_PEROXISOME	0.132034	0.017083	1.48572	0.138082	0.233013	−5.56224
HALLMARK_WNT_BETA_CATENIN_SIGNALING	0.139712	−0.01358	1.427229	0.154235	0.239908	−5.6458
HALLMARK_P53_PATHWAY	0.118958	−0.00521	1.407697	0.159939	0.239908	−5.67296
HALLMARK_GLYCOLYSIS	0.12231	−0.02451	1.114532	0.26567	0.362097	−6.03604
HALLMARK_ANDROGEN_RESPONSE	−0.09161	−0.01368	−1.08559	0.278264	0.362097	−6.06734
HALLMARK_INFLAMMATORY_RESPONSE	−0.05291	0.00149	−1.07801	0.281631	0.362097	−6.0754
HALLMARK_COMPLEMENT	−0.05119	0.006593	−0.93414	0.35075	0.41205	−6.21774
HALLMARK_ESTROGEN_RESPONSE_LATE	−0.09946	0.007701	−0.93365	0.351006	0.41205	−6.2182
HALLMARK_REACTIVE_OXIGEN_SPECIES_PATHWAY	0.068331	−0.02072	0.803555	0.422095	0.474857	−6.32939
HALLMARK_ANGIOGENESIS	−0.06601	0.01222	−0.76906	0.442275	0.477657	−6.35609
HALLMARK_MYOGENESIS	−0.06866	−0.02249	−0.62445	0.532663	0.55315	−6.4553
HALLMARK_NOTCH_SIGNALING	−0.05143	−0.02244	−0.5239	0.600614	0.600614	−6.51217

### Immune Cell Patterns in Molecular HNC Subclasses

**Figure 11:**
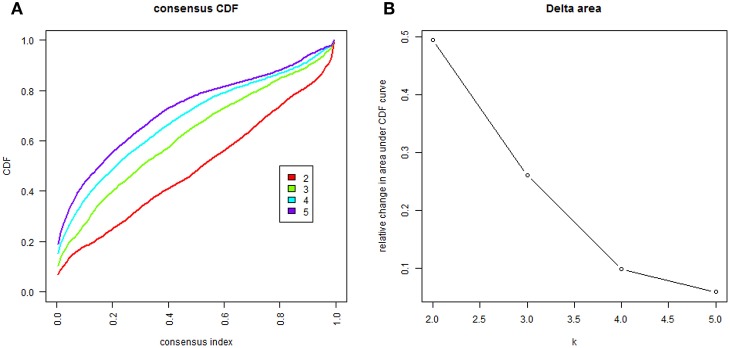
The cluster counts evaluated. **(A)** CDF curve of *K* = 2–7. **(B)** The relative change in area under the CDF curve of *K* = 2–7.

**Figure 12:**
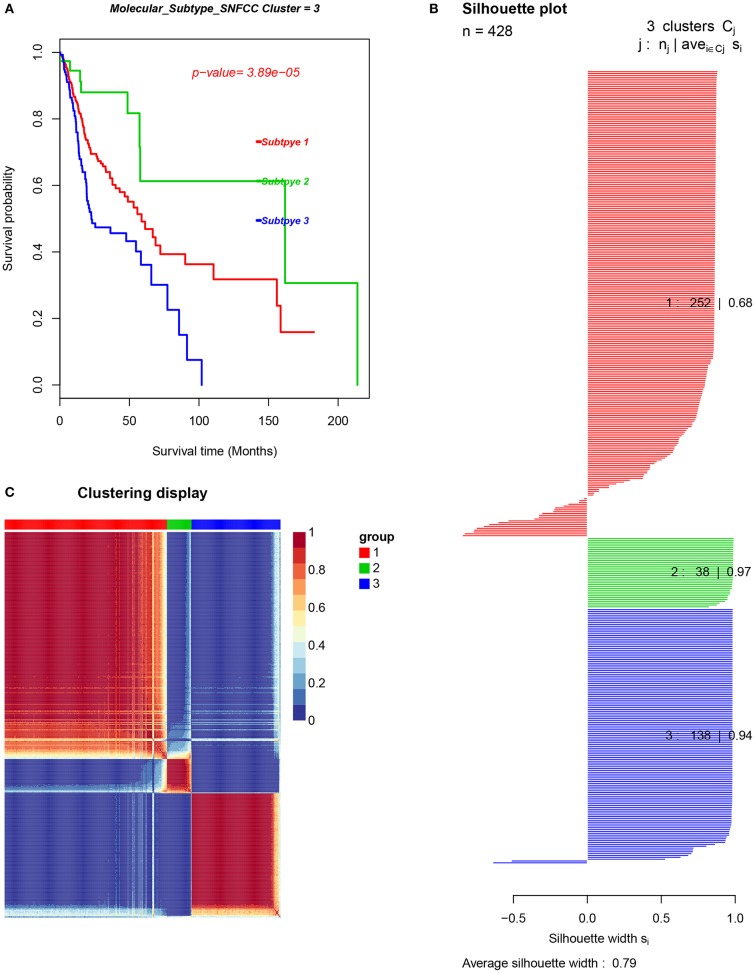
The cancer subtypes using SNFCC+ algorithm. **(A)** Log-rank test *p*-value for Kaplan–Meier survival analysis, **(B)** clustering heatmap visualizing the degree of the partitioning of the sample clusters, **(C)** average silhouette width representing the coherence of clusters.

In order to uncover different immune cell population infiltration of TME in HNC, molecular classification of HNC was identified by performing unsupervised consensus clustering of all tumor samples. The optimal number of clusters was determined by the *K* value. After assessing the relative change in the area under the cumulative distribution function (CDF) curve and consensus heatmap, we selected a three-cluster solution (*K* = 3), which has no appreciable increase in the area under the CDF curve (Figure 11). This finding resulted in 252 patients (59%) in cluster I, 38 patients (9%) in cluster II, and 138 patients (32%) in cluster III for the HNC cohort. The consensus matrix heatmap revealed the Cluster I and Cluster II, Cluster III well appeared individualized clusters in Figure 12A. The sample of each cluster was shown in Figure 12B. Moreover, clusters were associated with distinct patterns of survival in Figure 12C. Compared with Cluster I and Cluster III, the patients who were classified as Cluster II had a good prognosis (*P* < 0.001, log-rank test).

### Differentially Expressed Analysis of Genes/LM22 Immune Cell Fraction in Each HNC Subgroup

**Figure 13:**
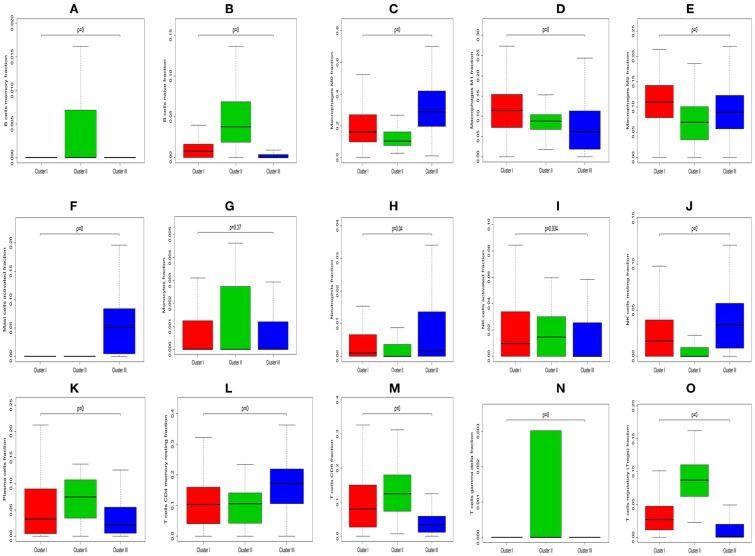
Box plot depicting the association between immune cell subset and three clusters, depicted *P*-values are from the Kruskal-Wallis tests. **(A–O)** Is for B cells memory, B cells naïve, Macrophages M0, Macrophages M1, Macrophages M2, Mast cells activated, Monocytes, Neutrophils, NK cells activated, NK cells resting, Plasma cells, T cells CD4 memory resting, T cells CD8, T cells gamma delta, and T cells regulatory (Tregs), respectively.

The Kruskal-Wallis test was used to identify quantitative genes/LM22 immune cell significantly associated with each subtype. As for differential infiltration of LM22 immune cell in HNC TME, Cluster 1 was defined by high level of Macrophages M1 and Macrophages M2, Cluster 2 was enriched by B cells memory, B cells naive, Monocytes, Plasma cells, T cells CD8, T cells gamma delta, T cells regulatory (Tregs), NK cells activated and T cells follicular helper. While Cluster 3 was defined by the high level of Macrophages M0, Mast cells activated, Neutrophils, NK cells resting, and T cells CD4 memory resting (Figure 13).

**Figure 14:**
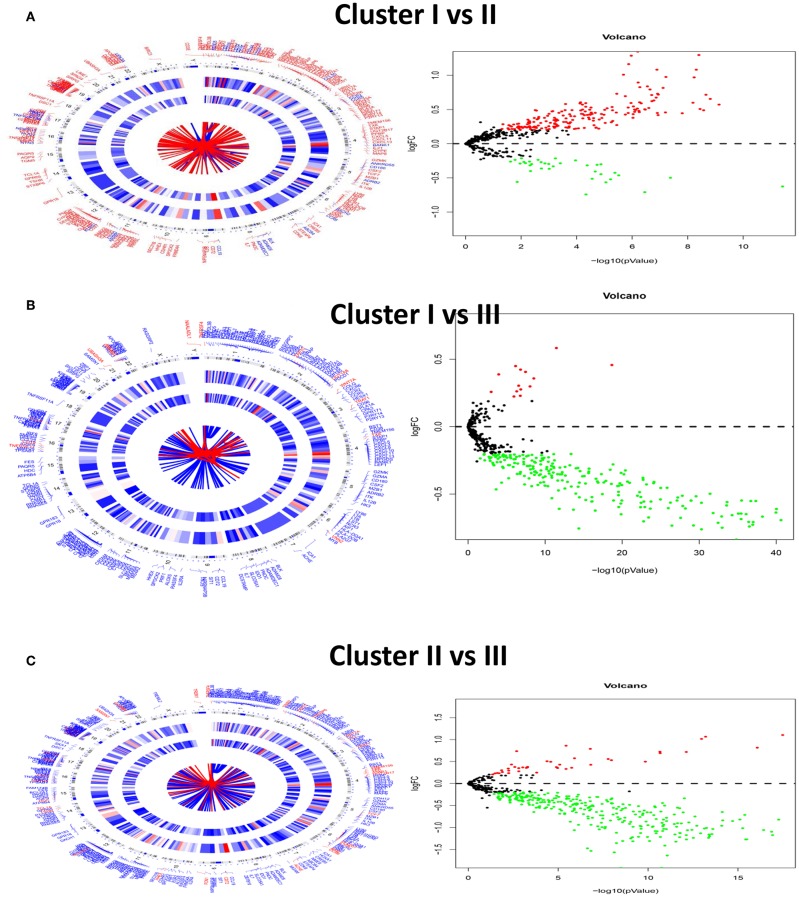
Circular visualization of chromosomal positions, hierarchical clustering, connectivity and severity gradients (Left) and a volcano plot (Right) in HNC subgroups. **(A–C)** Is for cluster I vs. Cluster II, cluster I vs. cluster III and cluster II vs. Cluster III.

In order to seek the molecular differences between HNC molecular subtypes and deriving subtype-specific biomarkers, the unpaired student *t*-test was used to identify quantitative genes significantly associated with each subtype. Gene differentially expressed analysis for unmatched subgroups was executed with the threshold of absolute log-fold change cutoff greater than 0.2 and FDR = 0.05. Figure 14 was shown by DEGs in concentric circles radiating among 3 Clusters. In subgroup 1 compared to subgroups 2, a total of 227 mRNAs (192 upregulated genes and 35 downregulated genes) were differentially expressed genes; In subgroup 1 compared to subgroups 3, a total of 242 differentially expressed mRNAs (14 upregulated genes and 228 down-regulated genes) were detected; In subgroup 2 compared to subgroups 3, a total of 333 differentially expressed mRNAs (41 upregulated genes and 292 down-regulated genes) were observed.

### Gene Functional Annotation and Gene Ontology (GO) Enrichment Analysis, GSVA Analysis for DEGs to Identify Molecular Subtypes

**Figure 15:**
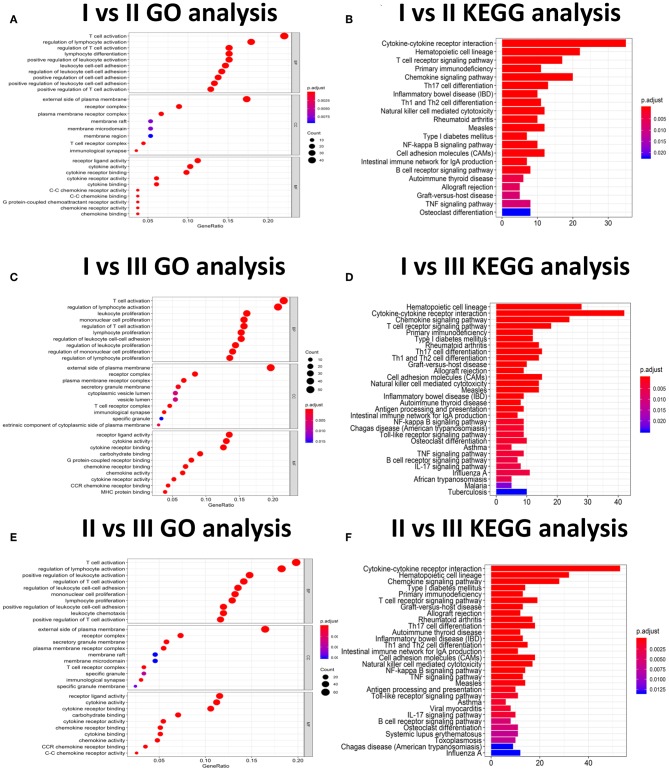
The GO and KEGG analysis for three HNC clusters. **(A–F)** Is for cluster I vs. Cluster II, cluster I vs. cluster III and cluster II vs. Cluster III.

In subgroup 1 compared to subgroup 2, a total of 533 GO terms of biological process, 8 GO terms of cellular component, and 53 GO terms of molecular function were identified to be significant (adjust *P*-value < 0.05). In subgroup 1 compared to subgroups 3, A total of 692 GO terms of biological process, 19 GO terms of cellular component, and 66 GO terms of molecular function were identified to be significant. In subgroup 2 compared to subgroups 3, A total of 676 GO terms of biological process, 19 GO terms of cellular component, and 55 GO terms of molecular function were identified to be significant. Top GO terms included cytokine activity, immune/inflammatory response, and chemokine activity (Figure 14). In addition, all the pathways that were yielded from the KEGG analysis were associated with immune response (Figure 15).

**Figure 16:**
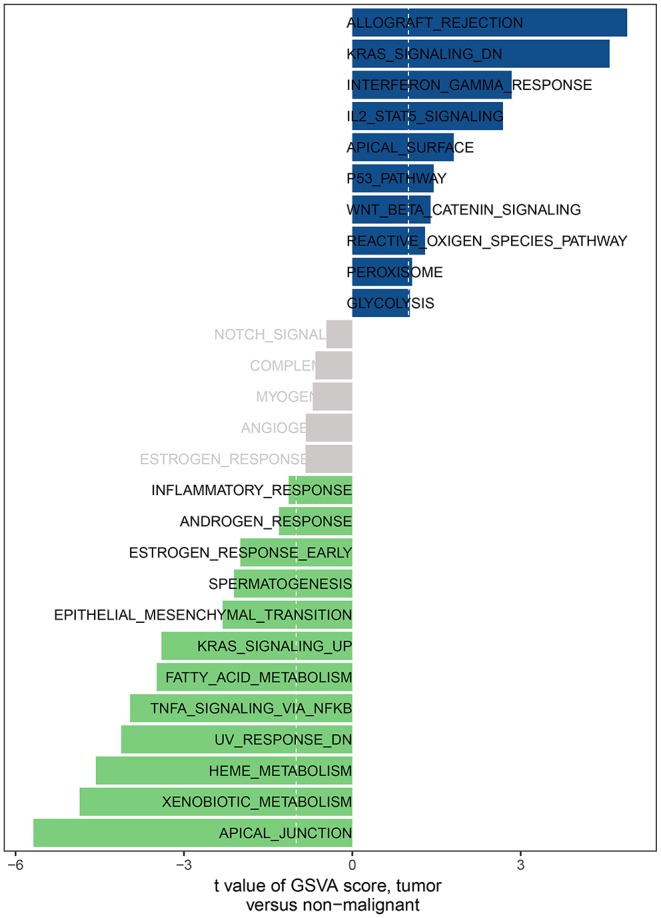
The GSVA analysis for three HNC clusters.

Gene set variation analysis with three clusters was analyzed by GSVA package of R software. The number of enriched pathways progressively increased from Subtype 1 through Subtype 3. The most significant gene sets enriched were ordered by significance (false discovery rate (FDR) *P*- and adjusted *P*-values) and listed in Table 4. As shown in Figure 16, several hallmark gene-sets, including “TNFA_SIGNALING_VIA_NFKB”, “APICAL_JUNCTION”, “EPITHELIAL_MESENCHYMAL_TRANSITION (EMT),” and “KRAS_SIGNALING_UP”, “ANGIOGENESIS”, were observed.

## Discussion

According to statistics from the National Central Cancer Registry of China (21) and the National Cancer Institute of USA (22), HSC is still the primary cancer disease worldwide. Especially in China, HNC is in the top ten causes of incidence rates and mortality in urban and rural regions in China (21). HNC is known for its rapid clinical progression and poor prognosis. The overall survival rate of HNC patients has not improved significantly over the past 20 years with the 5- year survival rate is ~45–50% (23). Therefore, HNC remains high-risk cancer affecting large numbers of people, threatening the lives of patients through local recurrence and metastatic cervical lymph nodes. Meanwhile, the potential molecular mechanism of HNC is not unknown and, more efforts should be used to this area to uncover the corresponding targeted therapy.

In current work, the CIBERSOFT was applied to evaluate the differential immune cell infiltration in paired HNC and adjacent normal tissue, and the results revealed that considerable difference in immune cell fraction occurred in both intra- and intergroup. Our work also uncovers the detail of infiltration of LM22 immune cell subsets in HNC that the proportions of macrophages account for more than 45%, in which 27% is M0, 9% is M2, and M1 make up 9%. In addition, T cells CD4 memory resting as the second proportion (12%) (Table 1). Compared to HNC and adjacent normal tissues, the proportions of Total Macrophage, Macrophages M0 and NK cells resting, total T cells, total B cells, T cells CD8, B cell navie, T cell follicular helper, NK cells activated, Monocyte, Mast cells resting produced statistical significance (*P* < 0.05). The total T cells, total B cells, T cells CD8, B cell navie, T cell follicular helper, NK cells activated, Monocyte, and Mast cells resting were increased in adjacent normal tissues when compared to the HNC tissues. In contrast, Macrophages M0 and NK cells resting were decreased in adjacent normal tissues. The mechanisms behind the activation of NK cells resting and Macrophages M0, deactivation of B cells naïve, T cells gamma delta, NK cells activated, and Mast cells resting in HNC remain unclear. Macrophages cells and NK cells have been detected in HNC (24, 25), we hypothesize that tumor cell-derived metabolites such as oxidized natural polyamines might be responsible for activation of Macrophages and NK cells and deactivation of Mast cells.

The prognostic importance of immune cell infiltration has been identified for various solid tumor types (26–28). In univariate Cox analysis, we found that B cells naive, T cells regulatory (Tregs), T cells follicular helper were associated with improved outcome, while Mast cells activated and Eosinophils were associated with poorer outcome. Eosinophils and Mast cells activated were associated with poorer outcome. To the best of our knowledge, the T- cell immune response is the primary event in antitumor immunity (29, 30), especially in HNC (31). The T and B cells are present in immune cell infiltrates of HNC and the degree of tumor-infiltrating T and B cells correlated with improved survival of cancers (32). Nevertheless, Eosinophils has been recognized to correlate with angiogenesis and metastasis (33) and increased Eosinophils proportions are related to poor prognosis. This immune phenotype was in line with a previous study (34). In addition, several immune cells are associated with clinical outcome. T cells follicular helper correlated with an increase in the level of GRADE stage. Total T cells and Macrophages cells, Neutrophils are associated with HPV status. Neutrophils and Macrophages M1 are related to radiation therapy. Our data further validated these findings from the previous studies that specific immune cells were related to predicting clinical outcome (35–37).

HNC can be robustly divided into three subtypes by SNFCC+ method. And the results demonstrated that three subtype classifications were significantly associated with patients' survival. Compared with Cluster I and Cluster III, the patients who were classified as Cluster II had a good prognosis. Compare with each Cluster of LM22 immune cell in HNC TME, Cluster I was defined by a high level of Macrophages cells infiltration, Cluster II was enriched by B cells infiltration and T cells infiltration. While Cluster III was defined by the high level of Mast cells infiltration, Neutrophils infiltration, and NK cells infiltration. Compared with each Cluster of DEGs, each cluster has its characteristic functional enrichment terms. Therefore, the three subtypes differed in overall survival, molecular characteristics.

In summary, our analysis of LM22 immune cell subsets using by CIBERSORT deconvolution algorithm provides the full information on immune cell landscape of HNC. Our finding has also uncovered a vital role in prediction for clinical outcome. In current work, this comprehensive assessment of LM22 immune cell infiltration models in TME shed light on how tumors respond to immunotherapy and might help clinicians to explore the development of the new drug.

## Data Availability Statement

The datasets analyzed in this study are available in The Cancer Genome Atlas (TCGA) public repository (https://cancergenome.nih.gov/).

## Author Contributions

JSo, DY, JZ, and ZD: wrote the main manuscript text. JSo and JL prepared Figures 1–16. JSu and JZ contributed to data analysis. All authors reviewed the manuscript.

### Conflict of Interest

The authors declare that the research was conducted in the absence of any commercial or financial relationships that could be construed as a potential conflict of interest.

## Supplementary Material

Supplementary Figure 1 The difference of immune infiltration between HNC the early stage (G1/G2) and the late stage (G3/G4) samples. **(A)** The total difference in immune cells infiltration. **(B)** Box plot of the distribution of CIBERSORT *P* value for T cells follicular helper.

Supplementary Figure 2 The difference of immune infiltration between HNC with radiation therapy and without radiation therapy samples. **(A)** The total difference in immune cells infiltration. **(B,C)** Box plot of the distribution of CIBERSORT *P* value for Macrophages M2 and Neutrophils, respectively.

Supplementary Table 1 Comparison of CIBERSORT immune fractions between grade G1/G2 and G3/G4.

Supplementary Table 2 Comparison of CIBERSORT immune fractions between with and without radiation therapy.
