# Supplementary figures and images for: Patterns of Immune Infiltration in HNC and Their Clinical Implications: A Gene Expression-Based Study
Source: Front Oncol. 2019 Dec 4;9:1285. doi: 10.3389/fonc.2019.01285 (PMC6904960; doi:10.3389/fonc.2019.01285)

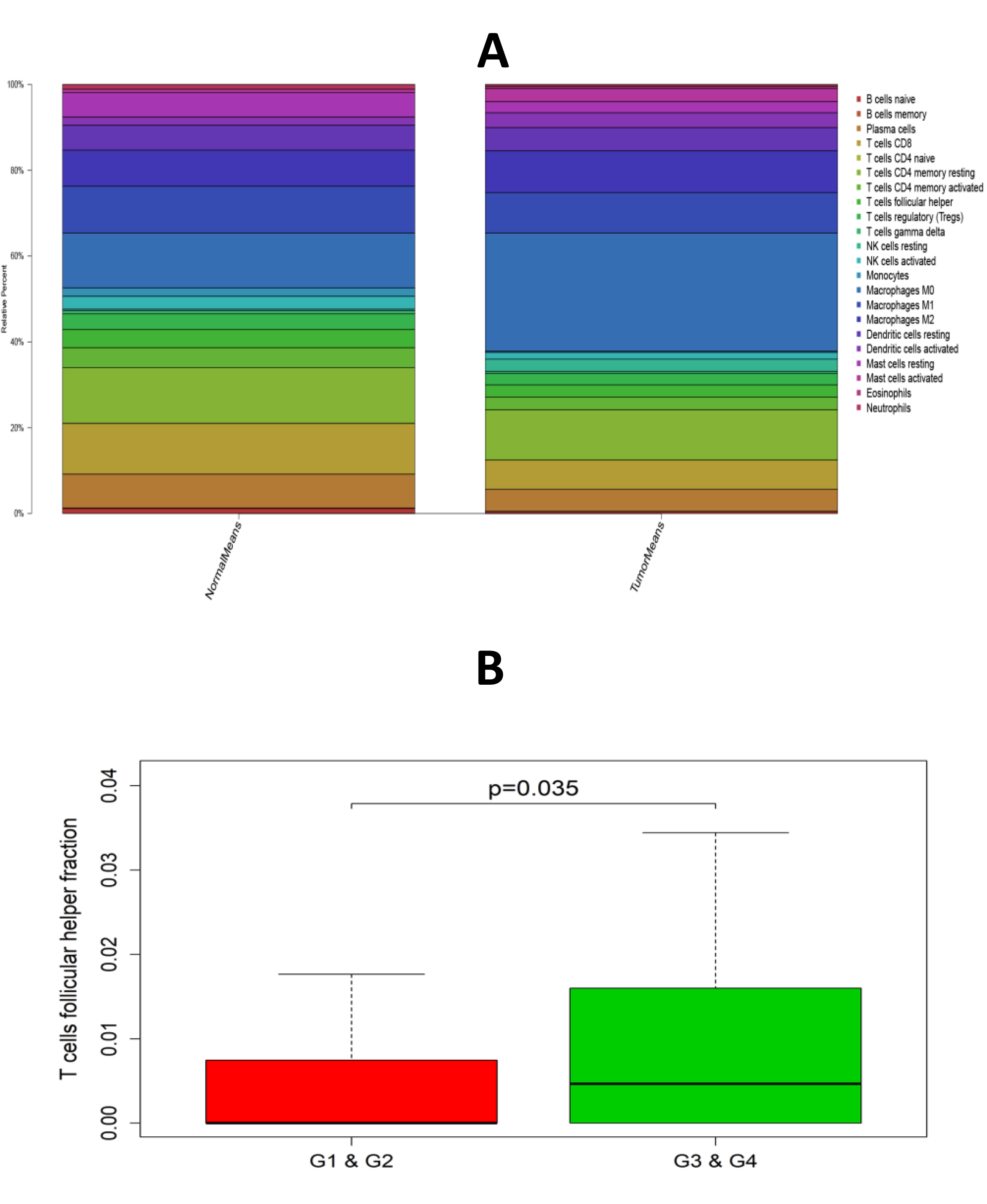

Supplement: Supplementary Figure 1 — The difference of immune infiltration between HNC the early stage (G1/G2) and the late stage (G3/G4) samples. (A) The total difference in immune cells infiltration. (B) Box plot of the distribution of CIBERSORT P value for T cells follicular helper. [file Image_1.tif]

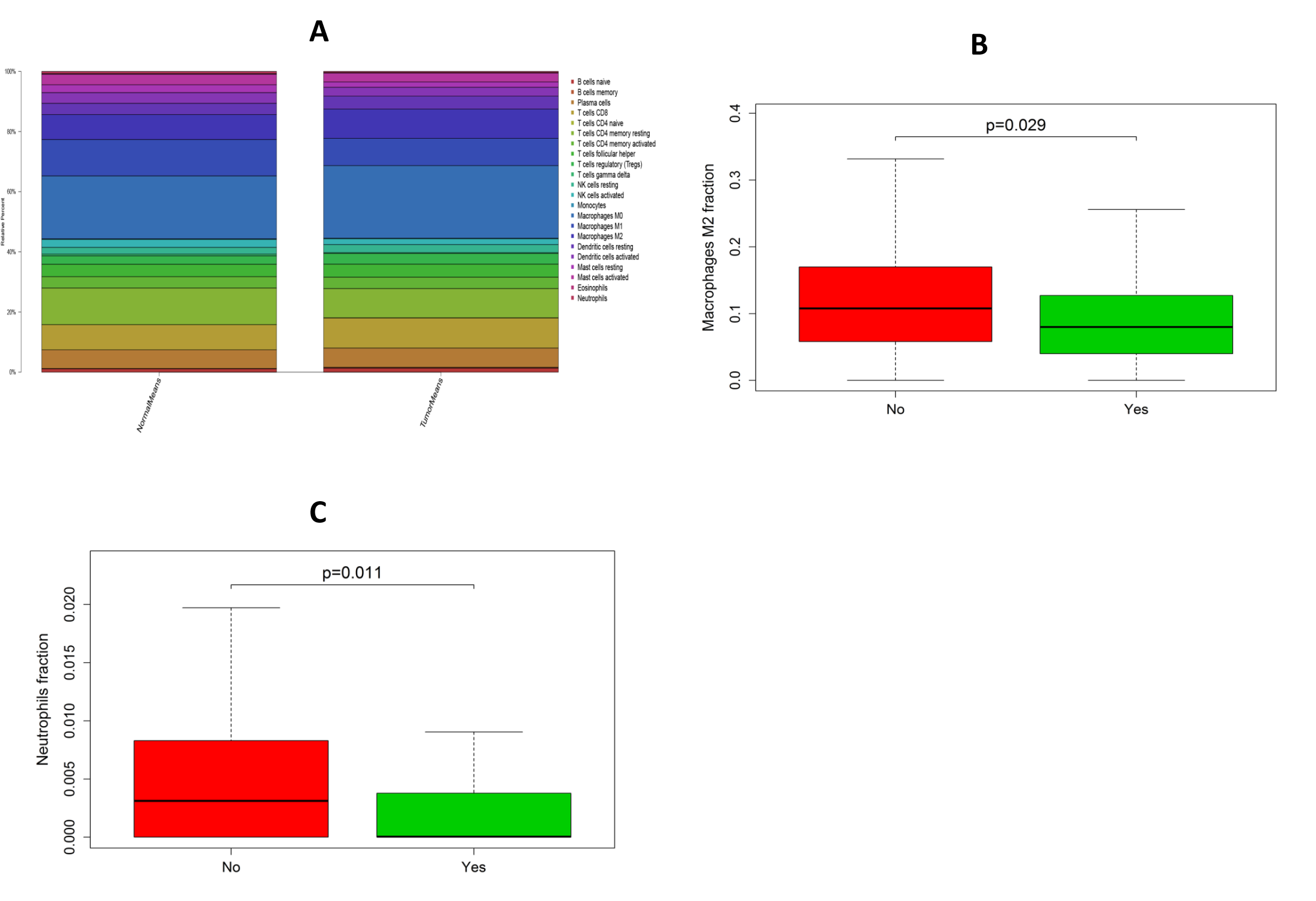

Supplement: Supplementary Figure 2 — The difference of immune infiltration between HNC with radiation therapy and without radiation therapy samples. (A) The total difference in immune cells infiltration. (B,C) Box plot of the distribution of CIBERSORT P value for Macrophages M2 and Neutrophils, respectively. [file Image_2.tif]
