# Supplementary material for: Patterns of Immune Infiltration in HNC and Their Clinical Implications: A Gene Expression-Based Study
Source: Front Oncol. 2019 Dec 4;9:1285. doi: 10.3389/fonc.2019.01285 (PMC6904960; doi:10.3389/fonc.2019.01285)
Supplement: Supplementary Table 1 — Comparison of CIBERSORT immune fractions between grade G1/G2 and G3/G4. [file Table_1.DOCX]

Supplementary Table 1 Comparison of CIBERSORT immune fractions between grade G1/G2 and G3/G4.

| Immune cell subtype | P value | normalMeans | tumorMeans | logFC |
| --- | --- | --- | --- | --- |
| B cells memory | 0.213238 | 0.012851 | 0.01501 | 0.224084 |
| B cells naive | 0.079433 | 0.062086 | 0.032478 | -0.9348 |
| Dendritic cells activated | 0.777916 | 0.010589 | 0.012809 | 0.274659 |
| Dendritic cells resting | 0.966732 | 0.051358 | 0.053964 | 0.071424 |
| Eosinophils | 0.990695 | 0.000627 | 0.000146 | -2.10338 |
| Macrophages M0 | 0.340643 | 0.184574 | 0.196985 | 0.093882 |
| Macrophages M1 | 0.058732 | 0.045509 | 0.060835 | 0.418736 |
| Macrophages M2 | 0.428673 | 0.159331 | 0.174782 | 0.133535 |
| Mast cells activated | 0.538015 | 0.001689 | 0.000494 | -1.77543 |
| Mast cells resting | 0.684631 | 0.058065 | 0.060485 | 0.058915 |
| Monocytes | 0.168005 | 0.028601 | 0.019199 | -0.57501 |
| Neutrophils | 0.386323 | 0.008586 | 0.009521 | 0.149173 |
| NK cells activated | 0.935319 | 0.018969 | 0.019641 | 0.050208 |
| NK cells resting | 0.777599 | 0.00306 | 0.003596 | 0.232834 |
| Plasma cells | 0.816848 | 0.03073 | 0.029169 | -0.07523 |
| T cells CD4 memory activated | 0.322819 | 0.009047 | 0.008353 | -0.11512 |
| T cells CD4 memory resting | 0.83501 | 0.175295 | 0.170014 | -0.04413 |
| T cells CD4 naive | 0.330012 | 0.000912 | 0 | inf |
| T cells CD8 | 0.845185 | 0.080883 | 0.080873 | -0.00018 |
| T cells follicular helper | 0.035115 | 0.006288 | 0.010026 | 0.673067 |
| T cells gamma delta | 0.051578 | 0.004534 | 0.001059 | -2.09788 |
| T cells regulatory (Tregs) | 0.38309 | 0.046417 | 0.040561 | -0.19456 |

Supplementary Table 2 Comparison of CIBERSORT immune fractions between with and without radiation therapy.

| **Immune cells subtype** | **P value** | **normalMeans** | **tumorMeans** | **logFC** |
| --- | --- | --- | --- | --- |
| B cells memory | 0.152686 | 0.000967 | 0.002734 | 1.499476 |
| B cells naive | 0.112119 | 0.011375 | 0.013703 | 0.268527 |
| Dendritic cells activated | 0.622732 | 0.035786 | 0.029636 | -0.27207 |
| Dendritic cells resting | 0.695065 | 0.037437 | 0.04299 | 0.199548 |
| Eosinophils | 0.997262 | 0.002014 | 0.001204 | -0.74237 |
| Macrophages M0 | 0.312242 | 0.208981 | 0.241187 | 0.206785 |
| Macrophages M1 | 0.128569 | 0.120981 | 0.090202 | -0.42355 |
| Macrophages M2 | 0.028923 | 0.082842 | 0.097571 | 0.23608 |
| Mast cells activated | 0.669489 | 0.035181 | 0.029886 | -0.23531 |
| Mast cells resting | 0.170196 | 0.02594 | 0.017615 | -0.55838 |
| Monocytes | 0.948586 | 0.002047 | 0.002439 | 0.253332 |
| Neutrophils | 0.011451 | 0.007335 | 0.003999 | -0.87528 |
| NK cells activated | 0.144386 | 0.026489 | 0.019165 | -0.4669 |
| NK cells resting | 0.431162 | 0.022092 | 0.026475 | 0.26112 |
| Plasma cells | 0.959187 | 0.061809 | 0.063787 | 0.045441 |
| T cells CD4 memory activated | 0.676692 | 0.0378 | 0.037727 | -0.00278 |
| T cells CD4 memory resting | 0.085848 | 0.121719 | 0.097379 | -0.32188 |
| T cells CD4 naive | 0.43817 | 0.000188 | 0.000544 | 1.531294 |
| T cells CD8 | 0.511307 | 0.08374 | 0.100059 | 0.256865 |
| T cells follicular helper | 0.916784 | 0.041138 | 0.043213 | 0.070975 |
| T cells gamma delta | 0.206927 | 0.005983 | 0.00329 | -0.86282 |
| T cells regulatory (Tregs) | 0.208891 | 0.028155 | 0.035195 | 0.321981 |
